# Cranial asymmetry arises later in the life history of the blind Mexican cavefish, *Astyanax mexicanus*

**DOI:** 10.1371/journal.pone.0177419

**Published:** 2017-05-09

**Authors:** Amanda K. Powers, Erin M. Davis, Shane A. Kaplan, Joshua B. Gross

**Affiliations:** Department of Biological Sciences, University of Cincinnati, Cincinnati, Ohio, United States of America; Laboratoire Arago, FRANCE

## Abstract

As a consequence of adaptation to the cave environment, the blind Mexican cavefish, *Astyanax mexicanus*, has evolved several cranial aberrations including changes to bone sizes, shapes and presence of numerous lateral asymmetries. Prior studies of cranial asymmetry in cavefish focused strictly on adult specimens. Thus, the extent to which these asymmetries emerge in adulthood, or earlier in the life history of cavefish, was unknown. We performed a geometric morphometric analysis of shape variation in the chondrocranium and osteocranium across life history in two distinct cavefish populations and surface-dwelling fish. The cartilaginous skull in juveniles was bilaterally symmetric and chondrocranial shape was conserved in all three populations. In contrast, bony skull shapes segregated into significantly distinct groups in adults. Cavefish demonstrated significant asymmetry for the bones surrounding the collapsed eye orbit, and the opercle bone posterior to the eye orbit. Interestingly, we discovered that cavefish also exhibit directional “bends” in skull shape, almost always biased to the left. In sum, this work reveals that asymmetric craniofacial aberrations emerge later in the cavefish life history. These abnormalities may mirror asymmetries in the lateral line sensory system, reflect a ‘handedness’ in cavefish swimming behavior, or evolve through neutral processes.

## Introduction

The development of symmetrical features is a fundamental biological process [1], stemming from axial patterning involving a complex network of genes and pathways [2]. Facial symmetry in humans contributes to subjective measures of beauty [3] and is hypothesized to be an indication of a healthy mate [4]. Symmetry is also an important factor in sexual selection with an association between female bias and symmetrical traits, such as a preference for symmetrical tail streamers in male barn swallows [5–6].

Despite constraints on symmetry in the natural world, a variety of taxa exhibit asymmetry [7]. Examples of normative asymmetries in body plan patterning include cerebral asymmetry in mammals [8–9], crossbill bird beaks [10], bee wing shapes [11], claws in male fiddler crabs [12], and jaw shape in scale-eating cichlid fish [13–14].

Bilateral asymmetries also appear to evolve in taxa despite the absence of obvious selective pressures. Examples of morphological asymmetry have been reported across a number of troglomorphic (“cave-dwelling”) taxa, including laterality in eye size reduction in the cave catfish [15–16], ornamental antennae in cave crickets [17], and mandibular structure in cave silverfish [18]. The etiologies of these asymmetries, as well as potential evolutionary pressures acting on these traits, remain unclear.

The blind Mexican cavefish, *Astyanax mexicanus*, demonstrates several bilateral cranial asymmetries manifested as left-right differences in the shape and patterning of the circumorbital bone series. These dermal bones, which encircle the eye orbit, demonstrate a variety of fragmentations and fusions [19–22], which are found in several independent cavefish populations. These phenomena have not been observed in closely related surface-dwelling morphs [22], providing an excellent comparative paradigm for understanding the developmental and molecular mechanisms contributing to cranial asymmetries.

Further, these morphological characters have only been evaluated in adult specimens when mature bone is present. To determine when, during the life history, cranial asymmetries are first manifested, we investigated the presence of cranial asymmetry in cavefish across ontogeny by evaluating chondrocranial shape in juvenile fish and the osteocranial shape in adults. In *Astyanax mexicanus*, chondrogenesis begins with the formation of the jaw at 3.5 days post fertilization (dpf) [23] and a cartilaginous skull persists until the mature osteocranium is formed by ~4 months [24]. The chondrocranium is of functional interest because it supports the early jaw [25] and can influence the shape and positioning of intramembranous bones formed later [26]. Furthermore, both tissues arise from contributions of the cranial neural crest [27] and cranial asymmetry in the juvenile chondrocranium may result from asymmetrical neural crest cell migration or differentiation. Alternatively, symmetrical cartilaginous shape may indicate that during late juvenile stages aberrant ossification processes cause cranial asymmetry in the adult. We tested these notions by implementing a geometric morphometric analysis of global shape symmetry [28–29] of the chondrocranium in juveniles (6–8 dpf) and the osteocranium in adults (1–3 years).

Despite early regression of the eye in cavefish, chondrocranial shape in juveniles is strikingly similar between surface fish and two different cavefish populations. In all three populations, the chondrocranium exhibited bilateral symmetry. However, adult osteocranial shape was both distinct and asymmetric in both cavefish populations. This indicates that, despite the regression of the visual system, the chondrocranial shape is conserved across early development in cave and surface morphs. Further, robust cranial asymmetries appear to emerge later in the life history. This work illuminates when during development cranial asymmetry emerges in cavefish, and focuses future studies on understanding how and why the left-right cranial axes are de-coupled in the wild.

## Materials and methods

### Animal husbandry and breeding

*Astyanax mexicanus* cave and surface fish were housed in a satellite aquatic facility at the University of Cincinnati (Cincinnati, OH, USA). All fish were maintained on an aquatic husbandry unit (Aquaneering, INC., San Diego, CA) that circulates reverse-osmosis water with a pH of 7.4 (±0.2) and conductivity of ~700μS (±50). System water is circulated through mechanical, bio, micron and UV filters. Adult fish were housed in 5- or 10-gallon glass tanks with separate flow control and reared under 12h light:12h dark photic schedule at a controlled temperature of 23 ± 2°C. Juvenile fish were reared in 5-gallon tanks with a heater (24°C) and an air bubbler until 8 days post fertilization (dpf). Food was administered daily in the morning with adults (1–3 years) receiving a slurry of dry flake food (TetraMin Pro) mixed with system water. Juveniles were fed live brine shrimp.

In this study, we analyzed Pachón cavefish from the ‘Asty-163 and 138’ pedigrees, which are relatives of fish originally collected from the Pachón cave (Sierra de El Abra region, Mexico). We also analyzed Tinaja cavefish from the ‘Asty-19’ pedigree, which are related to individuals collected from the Tinaja cave locality. Lastly, we analyzed surface fish from the ‘Asty-155 and -152’ pedigrees, which are relatives of individuals that originated from the Río Sabinas and Río Valls drainages near Ciudad Valles, Mexico. Adult fish were generously provided by Dr. Richard Borowsky (NYU). All breeding occurred between pedigree pairs at the University of Cincinnati. All experiments in this study were performed in accordance with the Guide for the Care and Use of Laboratory Animals of the National Institutes of Health and approved under protocol #10-01-21-01 by the UC Institutional Animal Care and Use Committee (IACUC).

### Analysis of chondrocranial shape

Juvenile fish were collected from Pachón cavefish (Asty-163 pedigree; n = 30), Tinaja cavefish (Asty-19 pedigree; n = 30) and surface fish (Asty-152 and -155 pedigrees; n = 30) populations between 6–8 dpf. Generally, microMRI has been used to study cartilaginous structures, but for landmark-based morphometric studies, this method would not yield sufficient resolution for three-dimensional (3D) landmarks [30]. Therefore, we combined chromatic clearing and staining with z-plane imaging to perform a two-dimensional (2D) shape analysis. All raw data collected from 2-D and 3-D analyses can be found in S2 Table.

Individuals were sacrificed using a lethal dose of 1% tricaine methylsulphonate (MS-222) anesthesia and fixed in 4% paraformaldehyde (PFA) for 2 hours at room temperature (RT). Specimens were then stained for cartilage according to procedures adapted from Potthoff (1984) [31]. Briefly, tissues were dehydrated in 50% EtOH overnight at RT and then 100% EtOH overnight at RT on an orbital shaker at low speed. Specimens were then placed in 3mL of 2% 8GX Alcian Blue for 2 hours at RT to stain for cartilage. Specimens were then treated with 3% H_2_0_2_ in 1% KOH to bleach melanic pigmentation, washed in 1% KOH, and stored in 30% glycerol and 70% KOH solution prior to imaging.

Following staining, specimens were imaged on a 2% agarose bed at 80x magnification for right and left sagittal plan imaging. Images were collected using the montage function (Leica Application Suite software v3.8, Wetzlar, Germany). Images were collected under identical illumination conditions and camera settings (brightness = 60%, saturation = 1.25 and automatic exposure).

**Fig 1:**
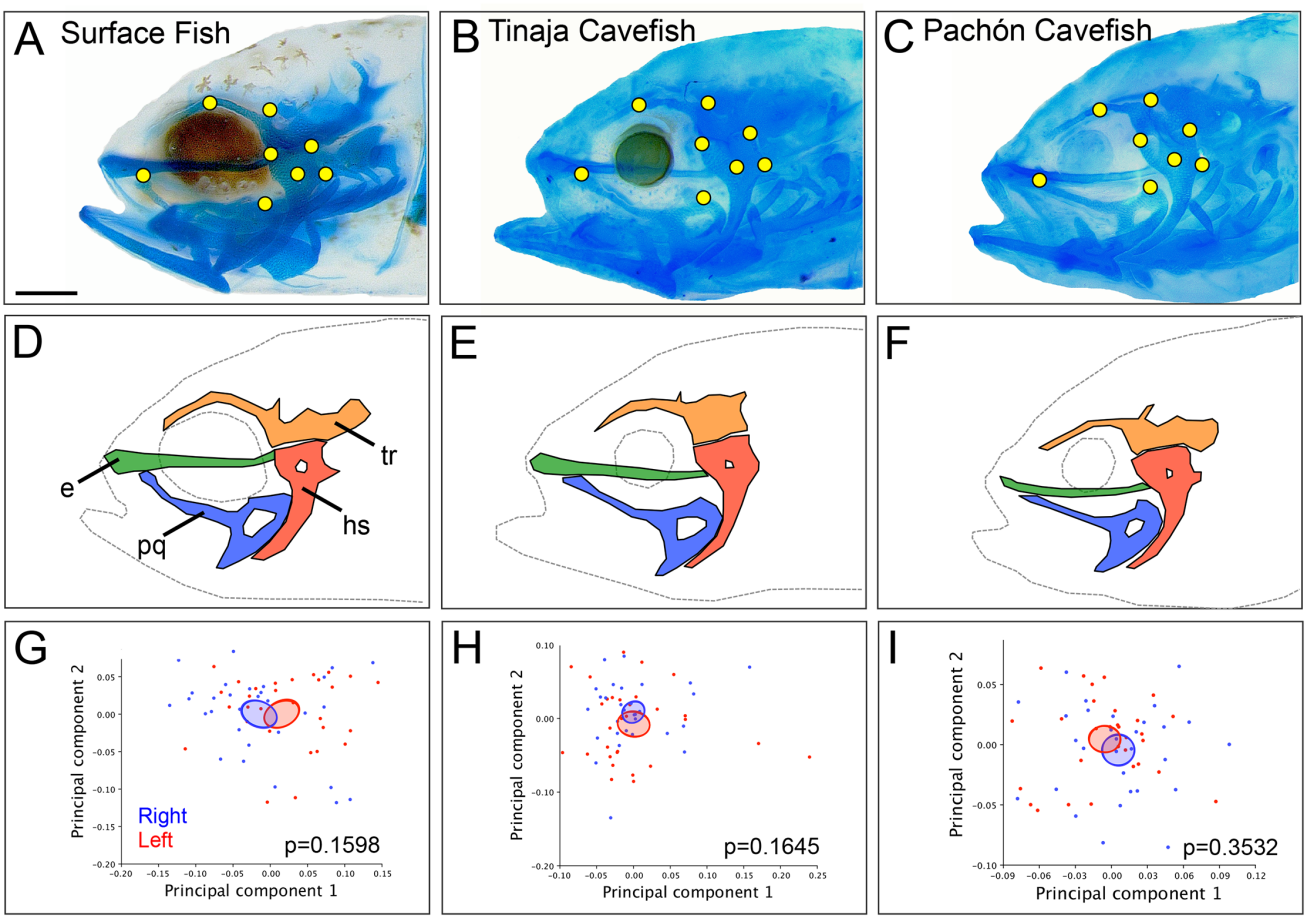
Chondrocranial shape is bilaterally symmetric in juvenile *Astyanax mexicanus*. Representative juvenile (8 dpf) specimens from surface fish (A), Tinaja (B) and Pachón (C) cavefish populations stained with Alcian blue and imaged at 80x magnification (scale bar = 1mm). Eight homologous landmarks (yellow; A-C) were placed on the ethmoid (e; green), trabecula (tr; orange), hyosymplectic (hs; red), and palatoquadrate (pq; blue; D-F). A Principal Components Analysis was used to compare the average shape of each individual on the right (blue) and left (red) sides for surface fish (G), Tinaja (H) and Pachón (I) cavefish. Confidence ellipses set to a significance threshold of p<0.05 overlap between the right (blue) and the left (red) shape for each population suggests shape quantification based on these landmarks is bilaterally symmetric. A student’s t-test revealed no significant differences in Procrustes distance between right and left sides for surface fish (p = 0.1598), Tinaja (p = 0.1645) and Pachón (p = 0.3532) cavefish.

**Fig 2:**
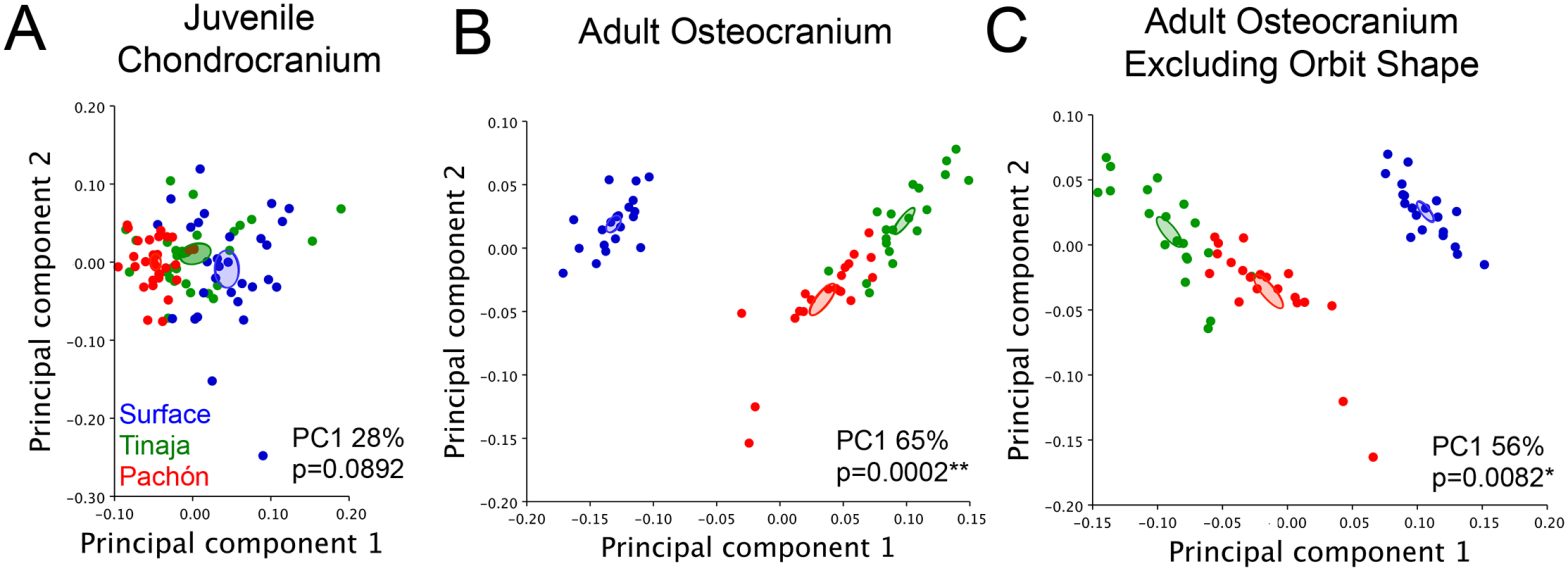
Juvenile constraint of chondrocranial shape contrasts adult osteocranial shape segregation in *Astyanax mexicanus*. Global chondrocranial shape was compared in n = 30 Pachón (red), Tinaja (green) and surface (blue) populations using a principal component analysis (A). Populations did not significantly differ from one another for juvenile chondrocranial shape (Procrustes ANOVA; p = 0.0892; PC1 28%). Analysis of global skull shape in n = 20 adults revealed divergence in shape between cave and surface fish populations (p = 0.0002; PC1 65%) (B). When the landmarks surrounding the eye were removed from the analysis, cave and surface populations were also segregated (C), suggesting global shape differences in the adult cranium are not strictly associated with eye regression and orbital collapse (p = 0.0082; PC1 56%). Confidence ellipses for each PCA were set to p<0.05.

**Fig 3:**
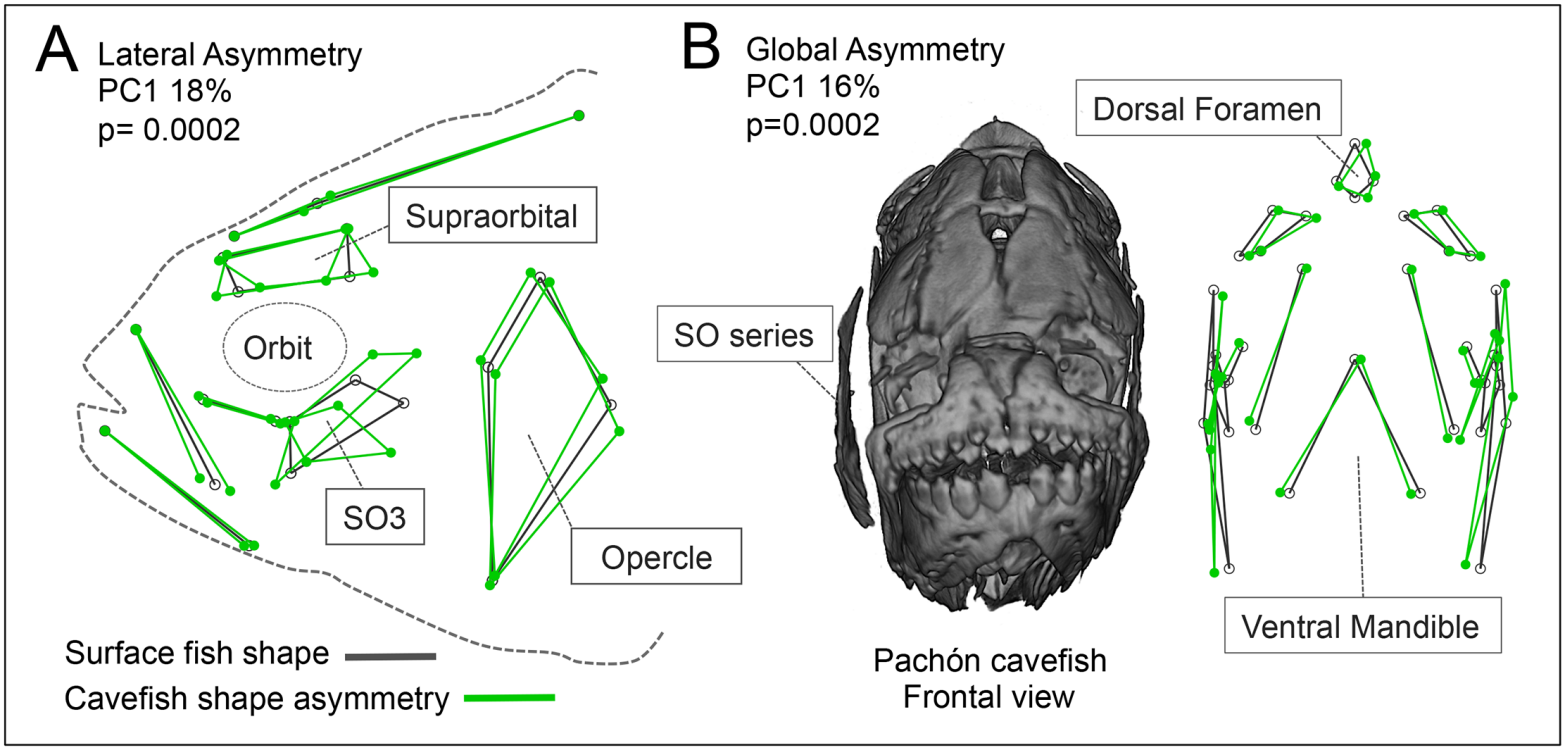
Adult cavefish exhibit fluctuating asymmetry in osteocranial shape. Cavefish demonstrate lateral asymmetry compared to surface fish (A; p = 0.0002). Lateral shape differences in PC1, accounting for 18% of shape asymmetry, occur in the supraorbital, SO3 and opercle bones (wireframe graphs in gray = average surface fish shape, and green = average cavefish shape). Cavefish demonstrate global 3D asymmetry as demonstrated in the frontal view of a Pachón cavefish (B; p = 0.0002). Shape differences in PC1, accounting for 16% of variation, indicate presence of fluctuating asymmetry in the shape of the osteocranium in cavefish.

To analyze lateral shape, 8 two-dimensional landmarks were digitized on both the left and right sides of each specimen. Homologous points were determined based on cartilaginous structures reported in Piotrowski *et al*. (1996) [32]. Landmarks were placed at the intersection of the ethmoid plate and the pterygoid process (Fig 1A–1C; 1), the dorsal ridge of the trabecula (Fig 1A–1C; 2), the occipital arch (Fig 1A–1C; 3), surrounding the hyosymplectic (Fig 1A–1C; 4–6), on the hyosymplectic foramen (Fig 1A–1C; 7) and the posterior edge of the palatoquadrate (Fig 1A–1C; 8). Landmarks on Meckel’s cartilage were avoided because of variable movement of the lower jaw during the fixation. Two-dimensional (2D) landmarks were placed using the ‘point selection’ tool in ImageJ software (NIH, v10.2) to recover XY coordinates for 8 points on the right and left sides of the chondrocranium. Landmarks were collected in 3 trials by the same investigator [33] and each trial was compared using a correlation analysis (Microsoft Excel Mac 2011 v14.3.5) with the average value of R = 0.99988. This strong correlation indicates a low level of error in data collection between trials, and therefore XY coordinates were averaged for downstream shape analysis.

Symmetry of shape was analyzed in MorphoJ software [34] using matching symmetry (paired structures present as separate, mirrored copies on the left and right sides of the body) and object symmetry (the plane of symmetry passes through the landmark configuration). The Procrustes fit with reflection of shape mapped the right and left shape configurations together and Procrustes distance was used as a measure of shape asymmetry between the right and left sides [35–36]. A one-way t-test was used to analyze differences between left and right Procrustes distances (StatPlus v5.9.80). A principle component analysis (PCA) was used to visualize left and right shape data across all three populations (confidence ellipses set at the threshold of p = 0.05).

### Analysis of osteocranial shape

Adult fish (1–3 years old with an average of 4.7cm standard length) were collected from Pachón cavefish (Asty-138; n = 20), Tinaja cavefish (Asty-19; n = 20) and Surface fish (Asty-155; n = 20) laboratory populations. Fish were sacrificed using a lethal dose of 1% tricaine methylsulphonate (MS222) anesthesia, fixed overnight at 4°C in 4% paraformaldehyde (PFA) fixative, and stored in 50mL tubes in 10% neutral buffered formalin (NBF).

Micro-Computed Tomography (MicroCT) imaging was performed at the University of Cincinnati Vontz Core Imaging Laboratory (VCIL). MicroCT provides high resolution, 3D tissue visualization in small animals [37–38]. The Siemens Inveon Multimodality System was used to collect axial, coronal and sagittal x-ray images (DICOM format) that were reconstructed into a three-dimensional ‘volume rendered’ skull using Amira software (v6.0; FEI Company, Hillsboro, OR). Bone tissue was labeled by tissue density using the ‘label field’ tool in Amira and segmented to create a 3D surface. Landmarks were collected from 39 homologous points on both lateral sides and along the dorsal midline of the skull, including: the maxillary, SO2, SO3, supraorbital, opercle, and supraoccipital bones; as well as at the edges of the dorsal foramen of the frontal bone and ventral aspect of the dentary and retroarticular bones [22; 39]. All landmarks were collected during two trials by a single investigator (*R* = 0.998 between trials) [33] and X, Y, Z coordinates were averaged for downstream shape analysis.

3D geometric morphometric analysis provides a robust and comprehensive analysis of phenotypic variation compared to 2D by capturing landmark data from more regions, such as the midline plane [40–41]. Shape symmetry in 3D was analyzed using MorphoJ software [34]. Cranial shape was defined using object symmetry including lateral and midline (dorsal and ventral) landmarks. Procrustes ANOVA was used to quantify left-right differences in shape symmetry [42]. A principle component analysis (PCA) was used to visualize left and right shape data across all three populations (confidence ellipses set at the threshold of p = 0.05).

## Results

### Cavefish juveniles exhibit chondrocranial bilateral symmetry

A principal objective of this study was to determine if there is evidence for early chondrocranial asymmetry that prefigures observed asymmetric bony traits in adult cavefish. Eight bilateral landmarks of homologous structures surrounding the eye orbit (i.e., the ethmoid, palatoquadrate, hyosymplectic and trabecular bones; Fig 1A–1C) were used owing to their proximity to suborbital bones that develop by ~4 months post-fertilization [24]. Landmarks were placed on the right and left sides for 30 juvenile (6–8 dpf) individuals from each population.

To analyze shape symmetry, landmarks from one side were reflected to match the orientation of the other side. PCA plots were then used to visualize shape variation between right and left sides of each individual (Fig 1G–1I). A student’s t-test of the Procrustes distance for each individual revealed no significant differences between chondrocranial shape on the right and left sides for surface fish (p = 0.1598), Tinaja cavefish (p = 0.1645), and Pachón cavefish (p = 0.3532). Thus, this data revealed complete overlap of chondrocranial shape, based on confidence ellipses (p<0.05) of the right and left sides for all three populations (Fig 1). This suggests that earlier in the life history, bilateral symmetry is maintained (or constrained) in the *Astyanax* juvenile chondrocranium.

### Convergence of chondrocranial shape across different populations of juvenile *A*. *mexicanus*

Different populations of adult cavefish naturally exhibit craniofacial alterations [19–20; 43]. We sought to discover when during the life history these cranial asymmetries first appear. Cartilage plays a key role in shaping the cranium and positioning of dermal bones [26], particularly the suborbital series, which exhibit dramatic abnormalities in cavefish. Therefore, we analyzed early juvenile chondrocranial shape between cave and surface-dwelling fish using geometric morphometrics.

Chondrocranial shape was analyzed using eight, two-dimensional landmarks placed on both sides of the juvenile chrondrocranium (6–8 dpf). Lateral landmarks were combined from each side of the face to achieve complete cranial shape (S1 Table). Chondrocranial shape was compared using Procrustes superimposition and a Principal Components Analysis (PCA) [28; 36] in a surface fish population and two cave-dwelling populations (Pachón and Tinaja). The first Principal Component (PC1) accounted for 28% of the total shape variation across populations. A PCA plot with PC1 and PC2 as the axes was used to visualize differences in cranial shape based on population. While each population grouped closely together, confidence ellipses set to p<0.05 did not overlap (Fig 2A). Further, Procrustes ANOVA revealed that the chondrocranial shape did not significantly differ between juvenile surface and cavefish (p = 0.0892). Therefore, the juvenile chondrocranium in *Astyanax* cavefish from the Pachón and Tinaja and surface (epigean) fish are morphologically similar.

### Segregation of osteocranial shape across adult surface and cavefish

Prior analyses of cranial shape differences between cave and surface *Astyanax* [22] have included qualitative [19–20; 39; 43] and quantitative [21; 24; 44–45] measures derived from two-dimensional studies. In order to evaluate three-dimensional (3D) shape differences, we collected comprehensive micro-computed tomography (microCT) scans of 20 adult individuals from each of the three populations.

We analyzed global cranial shape in adult surface fish, Tinaja, and Pachón cavefish. We found complete and significant segregation of shape between surface fish and both cavefish populations (Fig 2B; p = 0.0002). The first principal component (PC1) for these analyses accounted for 65% of the total variation in shape across populations. This demonstrates that there is a substantially greater difference in adult osteocranial shape compared to juvenile chondrocranial shape (PC1 28%).

We next reasoned that some cave-specific cranial shape differences may arise as a secondary consequence of eye regression and subsequent orbital collapse. Therefore, we removed landmarks surrounding the eye (i.e. the frontal, supraorbital and SO3 landmarks), and excluded orbital shape from our analysis. As a result, we discovered cave-specific cranial shape differences that arise independently from eye regression in both Pachón and Tinaja cavefish populations (Fig 2C; PC 1 56%, p = 0.0082). The shape differences captured by PC1 occurred within the opercle bone, dorsal foramen, maxilla and in the lower jaw. This indicates that many shape-related components of the adult cavefish cranium arise independently (or indirectly) from eye loss, and are evolving differently in the Pachón and Tinaja cavefish populations.

### Fluctuating asymmetry of the osteocranium in adult cavefish

Prior studies revealed that cavefish exhibit both aberrations in cranial structure, as well as asymmetries of structure, compared to surface-dwelling morphs [22]. For instance, SO3 fragmentation can occur on the left, right, or both sides of the face with varying numbers of distinct elements (i.e., “fragments”). However, the overall area of individual SO3 bones between the left and right sides appear to be the same [45]. This suggests that, despite fragmentations and fusions, the circumorbital bones occupy the same space across the left-right axis. We therefore decided to evaluate if global shape is similarly conserved across the left-right axis by implementing three-dimensional morphometrics to capture global shape variation.

Towards this end, we set homologous landmarks distributed along the dorsal, ventral and lateral sides of the osteocranium in adult fish for three populations. Landmark-based wireframe graphs for PC1 (18% asymmetrical shape variation) in the lateral view revealed shape asymmetry in the SO3, supraorbital and opercle bones in cavefish (Fig 3A). Global shape asymmetry can also be observed in the frontal perspective (Fig 3B) with a wireframe graph for PC1 (16% asymmetrical shape variation), which illustrates shape asymmetry in the maxillary, and dorsal regions. In the frontal aspect, qualitative observations of asymmetry included protrusion of the suborbital bone series on the right side (see Pachón cavefish individual, Fig 3B). A Procrustes ANOVA analysis revealed significant fluctuating asymmetry (i.e., non-directional deviations from bilateral symmetry) for global cranial shape (p = 0.0002), dorsal shape (p = 0.0092) and lateral shape (p = 0.0002) in cavefish (Table 1). Although significant shape differences were observed between surface and cavefish in the mandible, no shape asymmetry was detected (p = 0.8504). These results indicate that fragmentation and fusion of individual suborbital bones exhibit fluctuating asymmetries in natural populations of adult cavefish.

**Table 1:** Statistical analysis of osteocranial symmetry in adult cavefish compared to surface fish.

Procrustes ANOVA						
**Global Shape**	Effect	Sum of squares	Mean square	df	*F*	*P*
	Individual	0.62527728	0.005582833	112	118.07	<0.0001***
	Side	0.00572668	0.00010605	54	2.24	0.0002***
	Ind*Side	0.00510673	4.72845E-05	108	0.66	0.9976
	Residual	0.44901912	7.16139E-05	6270		
**Dorsal Shape**	Effect	Sum of squares	Mean square	df	*F*	*P*
	Individual	0.3853471	0.010704086	36	24.78	<0.0001***
	Side	0.01890813	0.001112243	17	2.57	0.0092**
	Ind*Side	0.01468686	0.000431967	34	1.46	0.0409*
	Residual	0.59110451	0.000296293	1995		
**Ventral Shape**	Effect	Sum of squares	Mean square	df	*F*	*P*
	Individual	0.86795689	0.433978444	2	368.82	0.0027**
	Side	0.00005384	5.38416E-05	1	0.05	0.8504
	Ind*Side	0.00235333	0.001176664	2	0.67	0.5174
	Residual	0.20123547	0.001765224	114		
**Lateral Shape**	Effect	Sum of squares	Mean square	df	*F*	*P*
	Individual	0.69570606	0.007905751	88	203.37	<0.0001***
	Side	0.00427543	9.50096E-05	45	2.44	0.0002***
	Ind*Side	0.00349859	3.88732E-05	90	0.45	1
	Residual	0.44117334	0.000086965	5073		

***p<0.001,

**p<0.01,

*p<0.05

### Cranial bending in cavefish is directionally asymmetric

**Fig 4:**
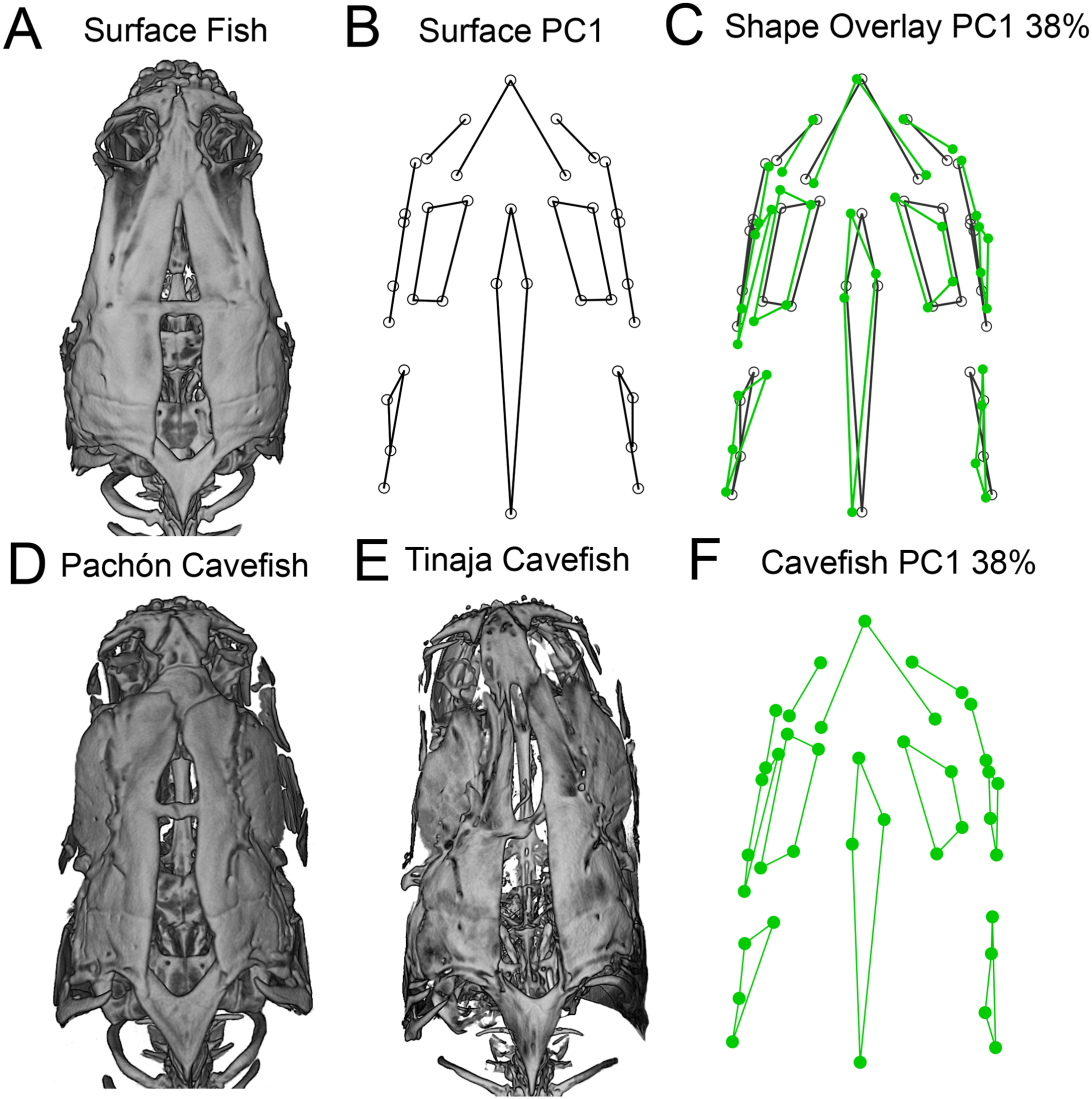
The dorsal osteocranium demonstrates directional asymmetry in adult cavefish. Surface-rendered microCT images of the dorso-cranium are presented for individual surface fish (A), Pachón (D) and Tinaja (E) cavefish. Landmark-based wireframe graphs for PC1 (capturing 38% of asymmetry shape variation) depict the average dorso-cranial shape for surface fish (B; black lines) and cavefish shape from both populations (F; green lines). Cavefish average shape (green) overlaid on surface fish average shape (black) reveals a dramatic dorso-cranial bend to the left (C). Procrustes ANOVA revealed significant and leftward-biased directional asymmetry in the cavefish dorsocranium (p = 0.0409). The bend in cavefish is most severe in the anterior dorso-cranium inclusive of the premaxilla, supraorbitals and the frontal bone, as well as the dorsal foramen, which extends posteriorly to the supraoccipital bone.

Two-dimensional morphometric analyses are typically limited to studying the lateral axis of the body, which lacks certain dimensions of shape [40]. MicroCT scanning allows three-dimensional rotation of specimens. During the assessment of microCT volume rendered images in 3D, we observed a frequent, and in some cases extreme, anterior-posterior bend in the dorsal cranium of adult cavefish from both the Pachón and Tinaja cave localities (Fig 4D and 4E). This dramatic dorso-cranial bend was never observed in surface fish skulls, and therefore is not a simple consequence of specimen fixation (Fig 4A).

Analysis of the landmarks on the dorso-cranium revealed a greater amount of shape asymmetry than in other regions of the skull (PC1 captured 38% of the shape asymmetry). Further, a wireframe graph of landmark configurations for PC1 underscores the symmetric shape of the dorso-cranium in surface fish (Fig 4B). In contrast, wireframe graphs for both cavefish populations revealed consistent leftward-biased shape asymmetry (Fig 4C and 4F), particularly in the supraorbital bones, dorsal foramen and maxilla. A Procrustes ANOVA revealed significant, leftward-biased, directional asymmetry of the cavefish dorso-cranium (Table 1; p = 0.0409).

## Discussion

### Conserved symmetry in chondrocranial shape in juvenile cave and surface fish

Despite shape differences in the adult skull, geometric morphometric analyses of juvenile chondrocranial shape revealed no significant differences between cave- and surface-dwelling fish. Further, the chondrocranium is bilaterally symmetric across juvenile *Astyanax* surface and cavefish populations. This early constraint on cranial shape and symmetry may stem from functional importance of the chondrocranium in critical early-stage feeding strategies. Powder *et al*. (2015) found evidence that early cranial development across different cichlid species was conserved, despite differences in adult skulls related to different feeding niches [46]. In *Astyanax* cavefish, the lateral palatoquadrate cartilage provides structural support until the maxilla is ossified [47]. Further, there is evidence that feeding kinematics differ significantly between 5 dpf (pre-ossification) zebrafish, exhibiting hyoid depression and cranial elevation, and in adult fish which exhibit abduction of bony elements during feeding [48]. Therefore, early shape convergence and symmetry in the juvenile chondrocranium of cavefish may be essential for survival until the skull is ossified.

**Fig 5:**
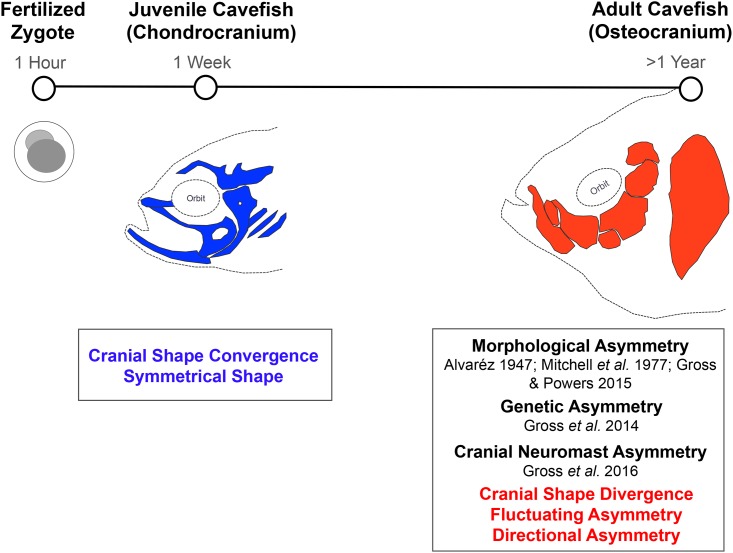
Timeline summary of cranial development in *Astyanax mexicanus* cavefish. The formation of the chondrocranium is complete by 6–8dpf. Cranial bones begin to develop around 20mm standard length, and the osteocranium is completely ossified by 35mm standard length. Previous studies reported morphological asymmetry [20–22] and genetic asymmetry [45] for individual cranial bones in adult cavefish. Gross *et al*. (2016) showed that cranial sensory neuromasts located on the suborbital #3 bone were more asymmetric in cavefish than surface fish [50]. Here, we demonstrate normal chondrocranial development, followed by divergence of cranial shape and fluctuating asymmetry at the level of individual bones, as well as directional asymmetry associated with dorso-cranial bending toward the left.

While it is clear that cartilage can affect the shape and positioning of cranial bones [26], the suborbital series of bones that exhibit asymmetrical fragmentations and fusions are intramembranous rather than endochondral. Thus, these bones are formed directly from cranial mesenchyme without an intermediate cartilaginous precursor [49]. This may indicate that the dermal bones undergo some aberrant developmental process, later in ontogeny, completely independent of chondrocranial formation. Lastly, eye regression is not as severe in early juvenile fish as it is in adult cavefish, which demonstrate a complete collapse of the eye orbit. This may explain, in part, why chondrocranial shape is conserved earlier, but later in the life history the osteocranial shapes differ dramatically between populations (Fig 5).

### Divergence in osteocranial shape across distinct populations of adult *A*. *mexicanus*

It has long been proposed that cranial differences between surface and cave *Astyanax*, including SO3 fragmentation, are the indirect result of eye regression [21]. Using experimental lentectomy, Yamamoto *et al*. (2003) found that certain cave-associated cranial features, including SO3 fragmentation, persisted in cavefish with surgically-induced eye development [24]. Conversely, when a cavefish lens was transplanted onto a surface fish embryo, resulting eye regression did not preclude suborbital bone fragmentation [24; 44]. Further, geographically- and phylogenetically-distinct cavefish populations undergo differing degrees and/or rates of eye regression [51–53]. To determine if the divergence of osteocranial shape was a consequence of eye loss in cavefish, we excluded landmarks directly surrounding the eye orbit. Cavefish continued to exhibit significant differences relative to surface fish in cranial shape not directly associated with eye loss. Therefore, these differences in global skull shape may instead occur as a consequence of other differences in behavior or morphology, e.g., feeding angle differences between the hypogean and epigean morphs [54–55]. Alternatively, differences in sensory and brain morphologies between the two morphs, such as larger olfactory bulbs, an elongated telencephalon and a reduction in the size of the optic tectum in cavefish [56], may influence osteocranial skull shape in adults.

### Fluctuating asymmetry in adult cavefish may be related to asymmetry in sensory neuromasts

At present, the developmental basis for cranial asymmetry in cavefish remains unknown. One possibility is that suborbital bone asymmetry in cavefish may be caused by the intrusion of dermal bone ossification beyond the suborbital canal into the collapsed eye orbit. As a result, more space may be created distally for bony condensations to spread during bone development. Alternatively, the formation of ectopic ossification centers coincident with an increase in the number of cranial neuromasts may influence abnormal bone development [24; 57]. Superficial neuromasts, a component of the lateral line system, are sensory structures associated with water flow, pressure and vibration detection in aquatic organisms [58]. Neuromasts have been proposed as ‘seeding centers’ for dermal bone ossification [59] and the altered number and position of neuromasts in the region of the SO3 bone may interfere with fusion of bony condensations, resulting in bone fragmentation. Gross *et al*. (2016) showed evidence of greater asymmetry in neuromast position on the SO3 in cavefish compared to surface fish (Fig 5) [50]. Therefore, one possibility is that asymmetry in the number and position of cranial neuromasts disrupts normative ossification leading to asymmetry in bony shape.

### Directional asymmetry in the dorso-cranial bend in adult cavefish and putative “handedness”

While directional biases have been observed in the cavefish cranium, the underlying causes remain unknown. Using traditional morphometrics, Mitchell *et al*. (1977) could discriminate between cavefish populations based on a metric for right-sided (but not left-sided) SO3 bone fragmentation [21]. More recently, Gross *et al*. (2014) discovered asymmetric genetic loci associated with right-sided SO1+2 fusion and SO3 fragmentation, whereas the genetic signal disappeared when traits were scored on the left side (Fig 5) [45]. This directional bias may indicate a possible “handedness” associated with craniofacial phenotypes in cavefish.

Many animals exhibit handedness and bilateral asymmetry in behavior and morphology, such as humans and great apes [60], asymmetry in vertical sensitivity patterns between ears in owls for prey localization [61], jaw laterality in scale-eating cichlids from Lake Tanganyika [14], and dolphins, which exhibit handedness in acoustic response [62].

The dorso-cranial bend observed in cavefish may reveal a cryptic handedness associated with lateral line-mediated and unidirectional “wall-following” swimming behavior. Cavefish swim continuously at high speeds, maintaining a parallel body orientation to walls surrounding their environment [63–64]. In contrast, surface fish show no evidence of entraining to the walls and vary their speeds and swimming direction, often remaining motionless in the darkest area of the tank [64]. This behavior is associated with differences in the lateral line system between the two morphs [58].

Cavefish produce larger and greater numbers of superficial neuromast organs on the lateral cranium compared to surface fish [58; 65]. Jeffery (2001) observed that cavefish have broader heads than surface fish, which may serve to increase the flow field, and augment neuromast sensitivity [43]. Additionally, ciliated hair cells that protrude from these neuromasts can produce unidirectional flow [66], which is consistent with unidirectional wall-following behavior in cavefish. As fish swim toward a wall, the stimulus to the cranial neuromasts significantly increased on the side of the body closest to the wall [67]. Further, orthogonal swimming creates a “blind spot” in hydrodynamic flow at the snout [63; 67], which may render the directionally asymmetric dorso-cranial bend phenotype adaptive for flow sensitivity in the absence of visual cues. For cavefish, a leftward-biased dorso-cranial bend may indicate a tendency for that fish to swim in the direction with the right lateral cranium facing the wall to achieve the greatest sensory input from surrounding structures. Because right-sided dorso-cranial bends in cavefish were observed only in rare occasions (n = 2/47 including both cavefish populations), the directional left-sided bias for the dorso-cranial bend may coincide with the predominance of right-handedness in humans.

Cranial neuromasts have also been implicated as sensory receptor organs mediating the cave-specific behavior of swimming toward a vibrating object, known as “vibration attraction behavior” (VAB) [58]. Interestingly, when the neuromasts located on the SO3 bone and within the eye orbit were experimentally removed, VAB was lost, suggesting that these particular neuromasts are critical for this cave-adapted behavior [58]. We observed that when cavefish exhibit the dorsal cranial bend of the skull, the suborbital bone series on the side opposite of the bend visibly protrudes (Fig 3B), which may accelerate hydrodynamic water flow over the neuromast region critical for VAB. While this hypothesis remains untested, it may provide evidence for a link between dorsal cranial asymmetry and asymmetry associated with SO3 fragmentation, as well as a possible evolutionary trade-off between aberrant cranial bone development and sensory system expansion in cave-adapted fish.

## Conclusion

This is the first analysis of craniofacial shape across life history in *Astyanax mexicanus*. Chondrocranial shape in early juveniles is conserved across distinct populations of cavefish and a surface fish population. Despite appreciable asymmetries in cavefish cranial bones, such as fragmentations and fusions of suborbital bones, the juvenile cavefish chondrocranial shape is bilaterally symmetric. This may indicate constraint during critical early stages of development related to feeding. In contrast to juvenile cranial shape, we identified substantial cave-specific differences in adult osteocranial shape. Both Tinaja and Pachón cavefish populations exhibited significant fluctuating asymmetry in global shape relative to surface fish. These asymmetries may be caused by, or contribute to, asymmetry in the number and position of cranial neuromasts in cavefish. Interestingly, the cavefish dorsal cranium demonstrates directional asymmetry with a leftward bias. This bend in the anterior-posterior orientation may provide an enhanced hydrodynamic flow, stimulating cranial neuromasts on one side of the head, and facilitating the unidirectional wall-following behavior unique to cavefish. Future studies will investigate the relationship between neuromast expansion and osteocranial asymmetry and the potential selective benefit to reducing the hydrodynamic imaging ‘blind spot’ by introducing a slight bend in the skull. Alternatively, in the perpetual darkness of the cave, relaxed selective pressures may ultimately explain the loss of symmetry in cranial phenotypes.

## Supporting information

S1 Table Position of landmarks used in morphometric analyses. The anatomical positions outlined for n = 39 three-dimensional landmarks on the adult osteocranium and n = 8 two-dimensional landmarks set on both sides of the chondrocranium in juvenile fish.
(XLSX)

S2 Table Raw dataset collected for morphometric analyses. The X and Y values at each landmark for individuals from each population (Surface, Tinaja and Pachón) were collected on both the right and left side of the cranium. The Procrustes distance for the two-dimensional cartilage analysis is shown for both the left and right sides for each specimen. The X, Y, and Z coordinates are shown for the each landmark placed for the analysis of the osteocranium.
(XLSX)
